# MycoMobilome: a community-focused non-redundant database of transposable element consensus sequences for the fungal kingdom

**DOI:** 10.1093/nargab/lqag026

**Published:** 2026-03-05

**Authors:** Tobias Baril, Daniel Croll

**Affiliations:** Laboratory of Evolutionary Genetics, Institute of Biology, University of Neuchâtel, Rue Émile-Argand 11, 2000 Neuchâtel, Switzerland; Laboratory of Evolutionary Genetics, Institute of Biology, University of Neuchâtel, Rue Émile-Argand 11, 2000 Neuchâtel, Switzerland

## Abstract

Transposable elements (TEs) are found in nearly all eukaryotic genomes. Despite significant advances in the sequencing of genomes, TE resources remain sparse, leading to a lack of traceability, reproducibility, and duplication of effort when annotating TEs. Here, we focus on the fungal kingdom and present MycoMobilome, a database of TE consensus sequences computationally curated using a set of 4309 genomes covering all major clades. The initial database contains 586 441 consensus sequences after filtering to remove putative host genes and low-quality consensus sequences. We provide a consistent naming convention to surface information on the confidence in the classification, including potential conflicting open reading frame functions, along with metadata to enable evaluation of TEs of interest and to determine whether further curation work is required on a case-by-case basis. Finally, we provide guidelines for community contributions and encourage researchers to deposit new or curated sequences, which will be incorporated into future MycoMobilome releases.

## Introduction

Annotation of transposable elements (TEs) is essential for the analysis of genomes, even if the focus is primarily on coding sequences. TE annotation relies on the initial discovery of TE families, termed *de novo* curation, followed by homology-based annotation using tools to recognize all members of each TE family. Various approaches exist for *de novo* curation, with the most common including RepeatModeler2 [1] and TEdenovo of REPET [2], whilst there are also databases providing previously curated TE families that can be used to annotate genomes, including Dfam [3, 4], TREP [5], RepBase [6], RepetDB [7], REXdb [8], and Yeast Transposons (https://github.com/bergmanlab/yeast-transposons) [9–11]. An exhaustive list of available databases is hosted at https://tehub.org.

The fungal kingdom spans almost a billion years of evolution, exhibits remarkable phenotypic and genomic diversity, and contains species found in nearly all ecological niches. The availability of genome resources for species spanning the kingdom is accelerating, partly due to efforts including the 1000 Fungal Genomes Initiative (https://1000.fungalgenomes.org) and broader efforts of the Darwin Tree of Life and Earth Biogenome projects [12, 13]. Despite these growing efforts, curated and multi-species TE resources for the fungal kingdom are almost non-existent. For example, in Dfam version 3.9, the only curated TEs are found for *Septoria linicola* and *Zymoseptoria tritici* (https://dfam.org/). Dedicated efforts to curate TE libraries for individual species include the plant pathogens *Magnaporthe oryzae* [14] and *Z. tritici* [15]. However, cross-referencing TE annotations across species has not been attempted, leading to reproducibility issues and duplication of effort as researchers perform annotations largely on a per-species basis.

Here, we present MycoMobilome (https://github.com/TobyBaril/MycoMobilome), a database of TE consensus sequences covering species across the fungal kingdom. We created MycoMobilome using all publicly available genome resources for fungi and provide researchers with a systematically generated TE consensus library with a persistent naming scheme to improve consistency and reproducibility. Consensus sequences are provided with labels to show whether their classification is supported by open reading frames (ORFs), and whether these consistently match the provided classification. Applying MycoMobilome classification to primary fungal models, we find that the genome fraction annotated as TEs increases by between +7.20% in *M. oryzae* to +13.41% in *Cryptococcus neoformans* compared to existing community TE annotations.

MycoMobilome is a key resource to facilitate investigations into TEs across the fungal kingdom and provides easy-to-implement methods for researchers to contribute new or updated sequences with attribution. Consistent application of MycoMobilome will improve consistency in TE naming conventions, whilst reducing the duplication of effort often accompanying repeat annotation in new genome assemblies.

## Materials and methods

### Initial curation for MycoMobilome

All publicly available genomes and associated proteomes for the fungal kingdom were sourced using Mycotools (version 0.32.3) [16]. Genomes were manually filtered to remove those under embargo, resulting in a final set of 4309 genome assemblies. Genome assembly metadata is provided in the MycoMobilome database (https://doi.org/10.5281/zenodo.18243921).

For each genome, a library of putative TE consensus sequences was generated using `earlGreyLibconstruct` in Earl Grey (v4.4.0) [17], configured with Dfam curated elements only (v3.7) [3, 4]. All 4309 *de novo* consensus libraries were subsequently combined into a single FASTA file containing 773 843 sequences. To generate the clustered non-redundant library, these sequences were clustered using the `easy-cluster` scalable cascaded clustering approach in MMseqs2 [18] with `–min-seq-id 0.8 -c 0.8 –cov-mode 1 –cluster-reassign` to cluster sequences to the 80-80-80 TE family rule [19], as adopted in very recent large-scale studies [20, 21], resulting in 354 315 non-redundant consensus sequences. The representative sequence for each cluster was extracted and labelled with the source genome (i.e. the genome from which the consensus sequence originated).

Autonomous TEs encode domains for their transposition activity, and the identity of these ORFs can be used to classify TEs [19, 22, 23]. Conversely, multicopy host proteins can erroneously be curated as repetitive sequences by automated TE curation methods, given their repetitive occurrence in host genomes. We translated all six frames of each TE consensus sequence using `transeq -clean -frame 6` in EMBOSS (v.6.6.0) [24] and identified matches to known host proteins present in the Fungi partition of RefSeq release 228 [25] using Diamond BLASTp (v 2.1.11) [26] with `–sensitive –matrix BLOSUM62 –evalue 1e-3`. Following this, we detected similarity to characterized TE proteins using two complementary approaches [20, 21]. First, we used HMMscan in HMMER (v3.4) [27] with `-E 10 –noali` to detect homology to known TE protein domain HMM models supplied in ProfilesBankForREPET_Pfam35.0_GypsyDB as part of the REPET software suite [2, 28, 29]. Hits were filtered to retain those with fseq_evalue ≤0.001 and fseq_bitscore ≥50. Next, we used BLASTp (v2.14.1+) [30] to detect homology to known TE protein domains found in RepeatPeps.lib, which is part of RepeatMasker (v4.1.9) [31] and is used to classify TEs using the RepeatClassifier module in the initial *de novo* curation step in Earl Grey. A minimum *e*-value of 1e-3 was defined, and nested hits were removed to retain the highest-quality protein hit for each query, followed by combining of adjacent and overlapping hits. Potential host gene hits were identified using the approach adopted by [20, 21, 32]. A TE consensus sequence was designated as a putative host gene if either: (i) there were hits to RefSeq queries and no hits to known TE queries; (ii) there were hits to both RefSeq queries and known TE queries, but at least 90 residues aligned to a RefSeq query did not overlap with alignments to known TE queries. Consensus sequences with no hits were retained in the TE library as putative non-autonomous TEs. In total, 24 571 consensus sequences were identified as potential host genes and removed.

To further refine the database, poor-quality TE consensus sequences were filtered. We define a poor-quality consensus sequence as <120 bp in length, as these are likely to be incomplete and poor quality. Further, majority-rule consensus sequences can sometimes contain unknown nucleotides, indicated with N in the sequence. We removed all sequences with ≥5% N using `seqtk comp` (https://github.com/lh3/seqtk). In total, 187 402 consensus sequences were removed from the unclustered library, and 53 103 consensus sequences were removed from the clustered library for not meeting the quality thresholds, resulting in final database sizes of 586 441 and 276 642 putative TE consensus sequences for the unclustered and clustered databases, respectively.

The majority of TE consensus sequences in publicly available databases remain uncurated. Despite this, these sequences remain a widely used resource. To enable the community to evaluate TE annotation quality of newly analysed genomes, we introduce an automated classification of each TE consensus against protein hits to known TEs (tables are provided in the MycoMobilome database). Each TE consensus was labelled with a two-letter code providing the level of evidence supporting the TE classification: `_PE` for protein evidence that matches the given classification, `_DA` for protein evidence that contradicts the given classification, and `_NE` for no protein evidence, which includes non-autonomous TEs.

No genome quality filtering was performed on our genome set to maximize taxonomic breadth. Consequently, some TE consensus sequences could be incomplete or low quality due to their curation in low-quality genomes. Consensus sequences in MycoMobilome that have been curated from genomes with an N50 below 50 kb and BUSCO score <90% complete are labelled with `LQGenome` in the header, enabling these to be indentified and filtered if required. In total, 18 768 (3.2%) consensus sequences in the unclustered library were generated from lower quality genomes, whilst 11 970 (4.3%) consensus sequences in the clustered library are represented by consensus sequences from lower-quality genomes.

Finally, each TE consensus sequence in MycoMobilome was given a unique name. MycoMobilome families are defined based on the clustered database, following the 80-80-80 rule: `MycMob1.1_family-[unique_family_number-six_letter_species_code]_[protein evidence]#[high_level_TE_classification]/[sub_level_TE_classification] @[genus species]`. For the unclustered database, the consensus sequences are labelled with a family ID consistent with the clustered database, and an extra member ID to discriminate between consensus sequences that come from the same cluster: `MycMob1.1_family-[unique_family_number]_member-[unique_member_number-six_letter_species_code]_[family_protein_evidence]#[high_level_TE_classification]/[sub_level_TE_classification] @[genus species]`.

### Comparison of existing TE annotation approaches with MycoMobilome

We wanted to assess the extent to which sampling TEs across fungal diversity enables improved detection of TE-derived genome content. To do this, we selected three well-studied organisms and compared TE annotation using MycoMobilome against the traditional approaches employed by the research communities working with each of these organisms. We compared our standardized approach with MycoMobilome against (i) annotation with known fungal TEs from RepBase and Dfam, as used in a recent study on *C. neoformans* [33]; (ii) a community annotation resource for *Candida albicans* (http://www.candidagenome.org/download/gff/C_albicans_SC5314/Assembly22/); (iii) annotation using a curated TE library with RepeatMasker for *M. oryzae* [14].

For *C. neoformans*, we adopted the same approach used in the original study and annotated the genome assembly for isolate CNA3 H99 (GCF_000149245.1) using RepeatMasker (v4.1.9) configured with Dfam (v3.9) and RepBase RepeatMasker Edition (release 20181026) and the options `-species fungi -norna -no_is`. For *M. oryzae*, we sourced the reference genome assembly MG8 (GCF_000002495.2) and annotated this with RepeatMasker using the TE consensus library provided with the publication [14]. For *C. albicans*, we obtained the genome assembly in FASTA format and the community feature annotation in GFF format for genome assembly SC5314 Assembly 22 from the Candida Genome portal (http://www.candidagenome.org/download/gff/C_albicans_SC5314/Assembly22/). To obtain a GFF of repeat regions, we filtered the feature GFF for features named long_terminal_repeat, repeat_region, and retrotransposon.

For comparison, we adopted our standardized approach as recommended with MycoMobilome. We annotated all three genome assemblies with `earlGreyAnnotationOnly`using Earl Grey (v6.3.2), providing MycoMobilome as the input library, with all other settings left as default.

Hits that are shared and unique to each methodological approach were identified using BEDTools intersect (v2.31.1) [34], and resultant feature coverage was calculated in R (v4.4.3) [35] with tidyverse [36]. For shared annotations, the width of the annotation in each case was calculated, rather than using a single length of annotation across compared methodologies, to account for variation in annotation size between methods. Venn diagrams were generated using the ggVennDiagram package [37].

## Results and discussion

### MycoMobilome assesses TE evidence across the fungal kingdom

The MycoMobilome release contains 276 642 consensus sequences, of which 39 265 have classifications supported by protein evidence. This provides a computationally curated resource for the diversity of TEs across the fungal tree of life. In particular, for clades without previous TE annotation efforts, this represents a very substantial expansion of genomic datasets. We performed filtering steps to stringently remove any consensus sequences with the potential to be host genes, as well as poor-quality consensus sequences. Following these efforts, we provide three versions of the database: a full database, a subset containing only TE consensus sequences with protein evidence, and a subset containing only TE consensus sequences lacking protein evidence. The compressed database files are provided via Zenodo (https://doi.org/10.5281/zenodo.18243921) in FASTA format, ensuring compatibility with a broad range of bioinformatics approaches. We provide guidance to non-specialists on how to best implement the MycoMobilome dataset into TE annotation pipelines (https://github.com/TobyBaril/MycoMobilome). We also provide a record of the genomes used to curate TEs across fungal diversity, and tables of hits to known TE proteins from multiple sources to cross-reference evidence supporting TE classifications for sequences of interest. To connect community-driven efforts and provide a centralized point for the genomics community, the unclustered consensus library has also been submitted to the Dfam consortium (dfam.org) [3, 4], where consensus sequences for species beyond fungi are also available.

### A community-based curation effort through continuous submissions

We also provide a MycoMobilome community (https://zenodo.org/communities/mycomobilome), hosted on Zenodo, with the aim of encouraging users to contribute new or improved (i.e. manually curated) TE consensus sequences to future releases. TE curation takes considerable work and expertise, which researchers should be recognized for. By adopting the Zenodo community approach, user contributions are assigned persistent DOIs, enabling contributors to be cited and recognized for their contributions to the wider genomics community. We provide guidance on how to contribute to MycoMobilome on the GitHub page, maximizing ease of use. Further, hosting such a resource on Zenodo reduces risks associated with longevity of the database. The nature of MycoMobilome as a database in FASTA format limits file sizes and enables efficient storage and reuse without extensive infrastructure or resource requirements.

### MycoMobilome improves TE detection in important fungal models

**Figure 1.**
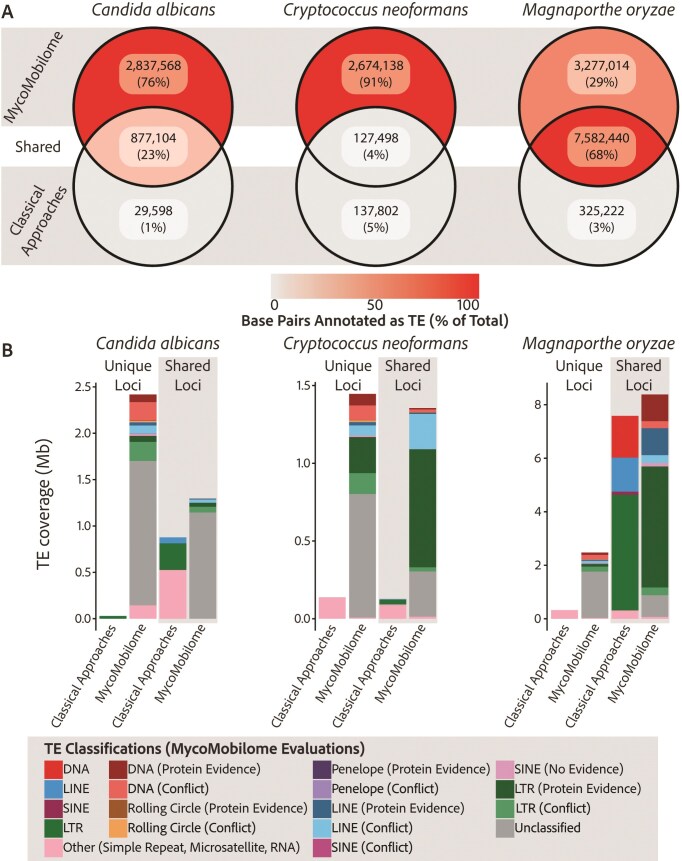
The use of MycoMobilome increases the fraction of genomes annotated as TEs in three key fungal pathogen models. (**A**) Venn diagrams illustrating the number of base pairs for each genome assembly that were annotated as TEs using either classical approaches or MycoMobilome (with Earl Grey). Depth of colour is proportional to the percentage of total base pairs annotated as TEs across all categories. Numbers in brackets show the percentage of all annotated base pairs that are found in each category, as labelled. (**B**) TE annotations split by approaches with which they are identified, and the classification of each annotated TE, as indicated in the key. Discrepancies in TE coverage at shared loci arise due to the defragmentation and overlap resolution steps automatically performed with Earl Grey following TE annotation with MycoMobilome. Supplementary Fig. S1 reports a re-analysis of the genomes using only TE coverage with protein evidence.

By sampling TEs across fungal diversity, we increase the proportion of well-studied host genomes that is attributed to TEs (Fig. 1 and Supplementary Table S1). For the major rice and wheat pathogen, *M. oryzae*, which has a well-curated TE library and much attention focused on TE dynamics, MycoMobilome increases the proportion of the host genome annotated as TEs by 7.20% (2.95 Mb). The use of Earl Grey to annotate TEs also increases the proportion of the genome annotated as TEs for loci detected both in previous annotations and MycoMobilome by 10.57% (i.e. ‘shared’; 801.1 kb). The improved annotation is likely due to the incorporation of defragmentation and overlap resolution steps, consistent with previous observations [17]. To assess whether the increase in annotated TE content was driven by elements typically absent from manually curated databases, we re-annotated *M. oryzae* using only the subset of MycoMobilome containing TEs with detectable protein evidence. This resulted in a substantial reduction in total annotated sequence (54%; ∼5.18 Mb), affecting both annotations unique to MycoMobilome (50% reduction; 3.28–1.40 Mb) and those shared with the original annotation approach (45% reduction; 7.58–4.16 Mb). Despite this reduction, the relative composition of annotations changed only modestly, with MycoMobilome-unique annotations comprising 23% of the total compared to 29% in the full database, and shared annotations increasing from 61% to 69% (Supplementary Fig. S1).

When annotating the genome of *C. neoformans* with MycoMobilome, we find an increase of host genome proportion annotated as TEs of 13.41% (2.54 Mb) compared to the previous annotation, which relied on fungal TEs hosted in Dfam and RepBase (Fig. 1). The large increase observed in *C. neoformans* likely arises from the absence of fungal TE families in existing databases, rather than over-annotation by MycoMobilome. Supporting this, 33.9% of the TE copies annotated using MycoMobilome come from consensus sequences curated in *Cryptococcus* species. In addition, a similar pattern is observed in *C. albicans*, where annotation using MycoMobilome increases the proportion of the genome recognized as TE by 9.82% (2.81 Mb) compared to the current community resource based on within-species TE characterization. The increase in unclassified TEs likely reflects the annotation of previously undetected, lineage-specific TEs lacking curated homologs. The proportion of unclassified TEs will decrease as representation of fungal lineages in curated TE reference databases, including MycoMobilome, increases. Hence, MycoMobilome provides a substantial increase in annotated TEs even in comparatively well-studied fungal models, the magnitude of which is consistent with observed increases in annotated TE content in well-studied organisms following the development of Dfam [4], where annotated TE bases increased by 150 Mb (5.1% of total genome size) in the human genome (hg38), 144 Mb (5.5% of total genome size) in the mouse genome (mm10), 6.5 Mb (5.5% of total genome size) in the nematode genome (ce10), and 0.95 Mb (0.7% of total genome size) in the fruit fly genome (dm6).

TEs can persist in host genomes over long evolutionary time, over which they will experience mutation and degradation, leading to the accumulation of genomic fossils, challenging our ability to detect these sequences as TE-derived. However, in related lineages, these TE families may persist and be retained with higher levels of identity and at higher copy numbers, enabling their detection. By sampling TEs across deep evolutionary time and using this information to generate a non-redundant TE library, we were able to detect homology to TEs that may evade detection using single-species *de novo* approaches, which require high TE copy numbers to generate an initial consensus sequence. We show that the use of MycoMobilome increases the proportion of the host genome annotated as TEs, demonstrating the power of sampling TEs across the fungal kingdom, and over deep evolutionary time, to be able to detect TE-derived sequences across fungal diversity.

## Limitations and future directions

A high proportion of unclassified TEs is expected in fungal genomes when analysed across broad phylogenetic scales, reflecting the uneven and lineage-biased representation of fungi in curated TE reference databases. While manually curated TE libraries exist for several well-studied ascomycete and basidiomycete species, these resources are typically species-specific and capture only a subset of TE diversity when considered across fungal lineages, isolates, and evolutionary timescales. In MycoMobilome, unclassified elements therefore primarily reflect historic bias in TE curation towards focal taxa, rather than limitations of the annotation framework. As curated fungal TE families expand, consensus classifications will be iteratively re-evaluated and updated while maintaining stable MycoMobilome family identifiers to ensure consistency across versions.

A related limitation is that MycoMobilome relies on computationally curated TE consensus sequences, which enables broad taxonomic coverage but may introduce some misannotations. Post-processing steps, stringent quality filtering, and homology-based annotation mitigate this risk; however, future improvements through targeted manual curation of representative lineages and expansion of curated reference libraries will further enhance classification accuracy.

MycoMobilome prioritizes taxonomic breadth over strict genome assembly quality thresholds to capture the widest possible diversity of fungal TEs. Heterogeneity in assembly quality and lineage sampling may result in some fragmented or incomplete consensus sequences. Excluding lower-quality genomes, however, would disproportionately remove early-diverging lineages and poorly sampled clades, reinforcing existing biases in curated TE resources. By retaining these genomes and explicitly flagging sequences derived from lower-quality assemblies, MycoMobilome balances coverage with transparency.

*De novo* TE discovery pipelines, including RepeatModeler2, can generate truncated or chimeric consensus sequences, particularly in fragmented assemblies or complex repeat landscapes. MycoMobilome implements post-processing steps to reduce these issues. Similarly, clustering consensus sequences using the 80-80-80 rule provides a convenient but imperfect approximation of TE family structure: it may overmerge young families or oversplit ancient or highly diverged families, and performs poorly for some TEs, such as DIRS-like elements and non-LTR retrotransposons. To accommodate these limitations, MycoMobilome provides both clustered and unclustered versions of the database, enabling users to apply alternative thresholds or phylogeny-based approaches.

Automated host-gene removal presents a further trade-off, as fungal genomes contain domesticated transposases and TE-derived genes. MycoMobilome applies conservative filtering criteria to minimize host-gene contamination, which may result in exclusion of some genuine TE-derived sequences. This choice prioritizes annotation specificity while allowing excluded sequences to be reintroduced as curated TE and TE-derived protein resources expand. Consistent with this conservative design, independent similarity searches indicate that retained unclassified elements are not enriched for conserved host genes (Supplementary Table S2), but instead predominantly match unannotated genomic regions or uncharacterized transcripts, supporting their interpretation as bona fide yet poorly curated TE-derived sequence.

Finally, many fungal TEs lack detectable ORFs or conserved domains due to rapid degeneration, nested insertions, or high sequence divergence. MycoMobilome preserves all original classifications assigned by RepeatClassifier and does not reclassify sequences based on ORF evidence. ORF and domain detection is provided solely as flags and metadata to indicate supporting evidence, allowing users to assess classification confidence on a per-element basis. As curated fungal TE protein models and reference libraries improve, these flags are expected to become increasingly informative while maintaining reproducible classification assignments.

Collectively, these limitations reflect the current state of fungal genome resources and TE curation. MycoMobilome is designed as a dynamic, community-oriented database: it provides broad taxonomic coverage and transparent annotations while creating a foundation for iterative refinement as genome quality, TE curation, and classification methods continue to improve.

## Conclusions

Here, we introduce MycoMobilome, a database of TE consensus sequences for the fungal kingdom. We aim to provide this database as a key starting point to facilitate genomic investigations in lineages spanning the fungal kingdom, compatible with widely used TE annotation tools, including Earl Grey and RepeatMasker. Key features include consistent naming conventions, metadata on the quality of TE classifications, and an easy-to-follow tutorial for the community to process additional genomes. We welcome contributions to the MycoMobiliome database and provide citable recognition for submitted TE annotations. We encourage the fungal research community to engage and expand the database scope to the benefit of all.

## Supplementary Material

lqag026_Supplemental_Files

## Data Availability

The MycoMobilome database has been deposited in the Zenodo database under DOI 10.5281/zenodo.18243921 (https://doi.org/10.5281/zenodo.18243921). User guidance and documentation are hosted on GitHub (https://github.com/TobyBaril/MycoMobilome) and under the DOI 10.5281/zenodo.17473060 (https://doi.org/10.5281/zenodo.17473060).
