# Supplementary material for: MycoMobilome: a community-focused non-redundant database of transposable element consensus sequences for the fungal kingdom
Source: NAR Genom Bioinform. 2026 Mar 5;8(1):lqag026. doi: 10.1093/nargab/lqag026 (PMC12961425; doi:10.1093/nargab/lqag026)
Supplement: lqag026_Supplemental_Files [file lqag026_supplemental_files.zip › Supplementary Table legends.docx]

**Supplementary Table legends**

**Supplementary Table 1**: MycoMobilome re-annotation of transposable elements in three key fungal pathogen models (*Magnaporthe oryzae*, *Candida albicans, Cryptococcus neoformans*). References for previous annotation efforts are provided in the main text.

**Supplementary Table 2**: BLAST queries for all unclassified MycoMobilome consensus sequences against the eukaryotic nt database (nt_euk; release 2026-02-03). The table repoorts the 98 sequences (0.17%) showing matches to mRNA entries with >50% query and/or subject coverage. Among these, the majority (72 / 98; 73.47%) corresponded to computationally predicted partial mRNAs, subunits, or isolated domains rather than well-characterised host genes
