# Supplementary material for: MycoMobilome: a community-focused non-redundant database of transposable element consensus sequences for the fungal kingdom
Source: NAR Genom Bioinform. 2026 Mar 5;8(1):lqag026. doi: 10.1093/nargab/lqag026 (PMC12961425; doi:10.1093/nargab/lqag026)
Supplement: lqag026_Supplemental_Files [file lqag026_supplemental_files.zip › Supplementary_Figure1.pdf]

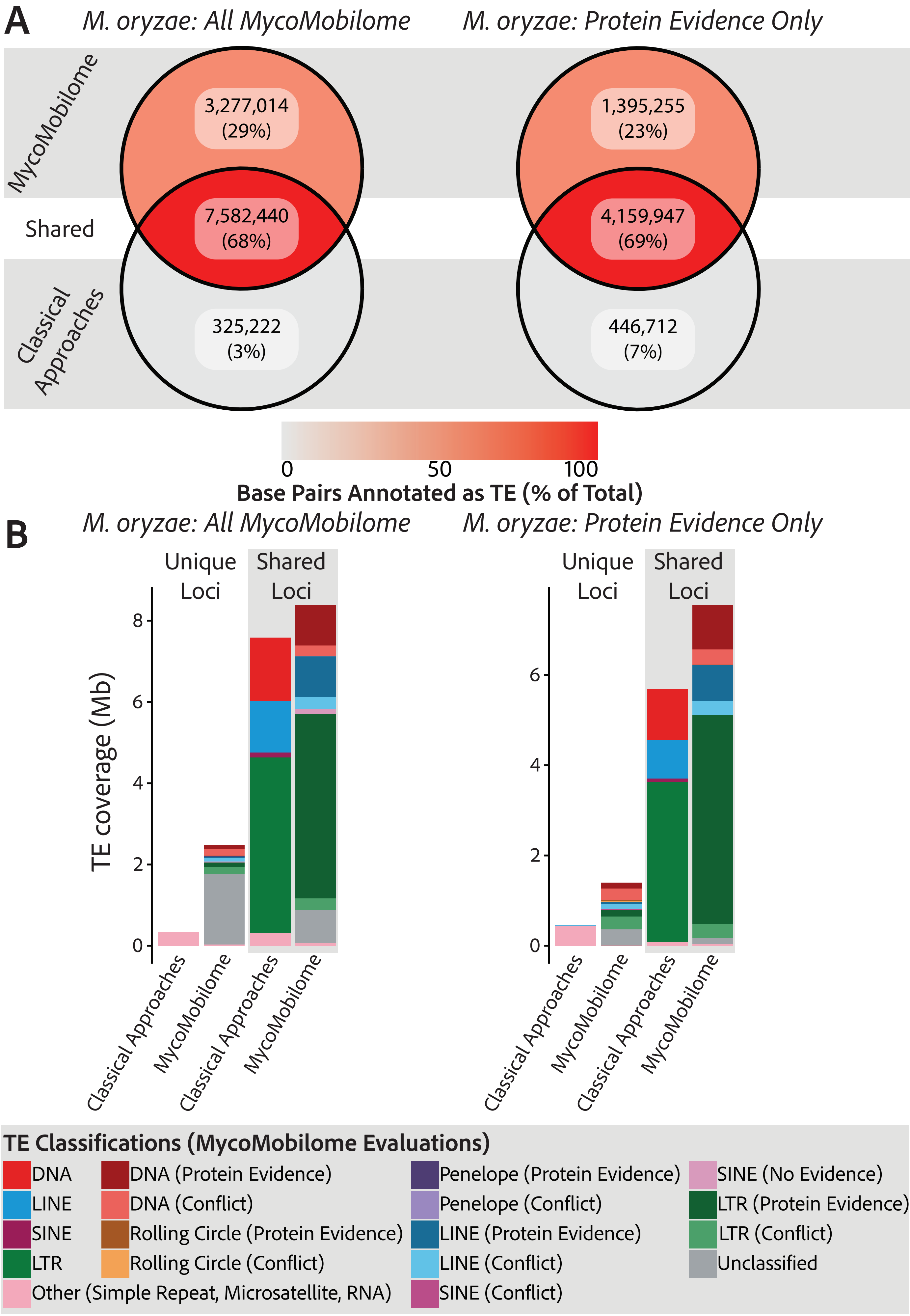

Supplemental Figure 1. Annotation of *M. oryzae* with the full MycoMobilome library, and the subset with protein evidence. **A.** Venn diagrams illustrating the number of base pairs for each genome assembly that were annotated as TEs using either classical approaches or MycoMobilome (with Earl Grey). Depth of colour is proportional to the percentage of total base pairs annotated as TE across all categories. Numbers in brackets show the percentage of all annotated base pairs that are found in each category, as labelled. **B.** TE annotations split by approaches with which they are identified, and the classification of each annotated TE, as indicated in the key. Discrepancies in TE coverage at shared loci arise due to the defragmentation and overlap resolution steps automatically performed with Earl Grey following TE annotation with MycoMobilome.
